# Neuroprotective Effects of PARP Inhibitors in *Drosophila* Models of Alzheimer’s Disease

**DOI:** 10.3390/cells11081284

**Published:** 2022-04-09

**Authors:** Anna Maggiore, Assunta Maria Casale, Walter Toscanelli, Ugo Cappucci, Dante Rotili, Maddalena Grieco, Jean-Philippe Gagné, Guy G. Poirier, Maria d’Erme, Lucia Piacentini

**Affiliations:** 1 Department of Biochemical Sciences “A. Rossi Fanelli”, Sapienza University of Rome, 00185 Rome, Italy; anna.maggiore@uniroma1.it (A.M.); maddalena.grieco@uniroma1.it (M.G.); maria.derme@uniroma1.it (M.d.); 2 Department of Biology and Biotechnology “Charles Darwin”, Sapienza University of Rome, 00185 Rome, Italy; assuntamaria.casale@uniroma1.it (A.M.C.); walter.toscanelli@uniroma1.it (W.T.); ugo.cappucci@uniroma1.it (U.C.); 3 Department of Chemistry and Technology of Drugs, Department of Excellence 2018–2022, Sapienza University of Rome, 00185 Rome, Italy; dante.rotili@uniroma1.it; 4 Department of Molecular Biology, Medical Biochemistry and Pathology, Faculty of Medicine, Laval University, Québec City, QC G1V 0A6, Canada; jean-philippe.gagne@crchudequebec.ulaval.ca (J.-P.G.); guy.poirier@crchudequebec.ulaval.ca (G.G.P.)

**Keywords:** Alzheimer’s disease, PARP-1 inhibitors, *Drosophila melanogaster*

## Abstract

Alzheimer’s disease (AD) is an irreversible age-related neurodegenerative disorder clinically characterized by severe memory impairment, language deficits and cognitive decline. The major neuropathological hallmarks of AD include extracellular deposits of the β-amyloid (Aβ) peptides and cytoplasmic neurofibrillary tangles (NFTs) of hyperphosphorylated tau protein. The accumulation of plaques and tangles in the brain triggers a cascade of molecular events that culminate in neuronal damage and cell death. Despite extensive research, our understanding of the molecular basis of AD pathogenesis remains incomplete and a cure for this devastating disease is still not available. A growing body of evidence in different experimental models suggests that poly(ADP-ribose) polymerase-1 (PARP-1) overactivation might be a crucial component of the molecular network of interactions responsible for AD pathogenesis. In this work, we combined genetic, molecular and biochemical approaches to investigate the effects of two different PARP-1 inhibitors (olaparib and MC2050) in *Drosophila* models of Alzheimer’s disease by exploring their neuroprotective and therapeutic potential in vivo. We found that both pharmacological inhibition and genetic inactivation of PARP-1 significantly extend lifespan and improve the climbing ability of transgenic AD flies. Consistently, PARP-1 inhibitors lead to a significant decrease of Aβ42 aggregates and partially rescue the epigenetic alterations associated with AD in the brain. Interestingly, olaparib and MC2050 also suppress the AD-associated aberrant activation of transposable elements in neuronal tissues of AD flies.

## 1. Introduction

Alzheimer’s disease (AD) is an irreversible neurodegenerative disorder that is considered one of the principal causes of age-related dementia and it is clinically described by a severe cognitive impairment in addition to language, and memory deficits. In the pathogenesis of AD, a pivotal role is played by two neurotoxic proteins that aggregate and accumulate in the central nervous system: amyloid-β (Aβ) peptide and hyperphosphorylated tau protein. Extracellular amyloid plaques, composed of Aβ peptide, and intracellular neurofibrillary tangles (NFTs), composed of hyperphosphorylated tau, are considered the major histopathological hallmark lesions of AD; they trigger a cascade of molecular events culminating in neuronal damage and cell death [1].

The aggregation of misfolded proteins leads to the overproduction of intracellular free radicals, which in turn, causes oxidative stress and cellular oxidative damage, tightly associated with the development and/or progression of neurodegenerative disorders such as AD [2,3].

Increasing evidence suggests that epigenetic mechanisms might play a crucial role in the development of AD (see [4] for a review). In particular, poly(ADP-ribosyl)ation (PARylation), catalyzed by a group of enzymes known as poly(ADP-ribose)polymerases (PARPs) [5], plays a central role in the molecular network of interactions responsible for AD pathogenesis [6,7,8,9,10,11,12,13,14,15].

PARP-1 is the founding member of the ADP-ribosyltransferases (ARTs) family and catalyzes a NAD^+^-dependent poly(ADP-ribose) (PAR) polymerization reaction onto amino acid residues of acceptor proteins. Several proteins are described as targets of PARylation, including core histones, histone H1 and a variety of nuclear proteins involved in gene regulation and chromatin remodeling [16,17]. Both PARP-1 and H1 are widely distributed across the genome and their depletion can promote large-scale alterations in chromatin structure [18,19,20,21]. They may alter nucleosome spacing and promote the compaction of nucleosome arrays [17,22,23,24]. PARP-1 and H1 compete for binding to nucleosomes and exhibit a reciprocal pattern of binding at actively transcribed promoters: H1 is depleted and PARP-1 is enriched [25]. By altering chromatin structure and destabilizing nucleosome organization, PARylation contributes to the histone code that regulates chromatin structure and gene expression. Moreover, PARP-1 modulates many other cellular processes important for the maintenance of cellular functionality and viability such as DNA damage repair, RNA processing, cell cycle regulation, proteasomal degradation, mitochondrial function, oxidative stress and aging [26,27,28,29,30,31,32].

One of the earliest studies of PARP-1 contribution to AD was carried out by Love and colleagues [6]. The authors observed an overactivation of PARP-1, with a concomitant increase of PAR levels, in the frontal and temporal lobe of the brains of AD patients. These results were subsequently confirmed with other experiments using both animal and cellular models where PARP activity was evaluated in the presence of Aβ peptide [7,33,34,35,36,37]. Several PARP-1-mediated mechanisms were proposed to promote the neurodegenerative process such as metabolic impairment related to NAD^+^ depletion and glycolysis arrest or different death pathways triggered by intracellular stress conditions and chronic inflammation [38,39,40,41,42,43,44,45,46].

Despite considerable efforts, the molecular mechanisms involved in the complex phenotype of AD are still largely unknown, and effective drug therapy is still not available to slow down or arrest the progression of the disease. Therefore, the investigation of new therapeutic targets that allow the prevention or delay the progression of the disease, constitutes an undeniable objective of great importance and interest, which ought to be considered an immediate priority.

The principal AD treatments approved by the Food and Drug Administration (FDA) are based on acetylcholinesterase inhibitors (AChEIs), including donepezil and rivastigmine that facilitate cholinergic transmission, and N-methyl-D-aspartate receptor (NMDAr) antagonists, such as memantine [47,48] that regulate glutamatergic transmission. However, both therapies have modest symptomatic effects and only slow the progression of the disease.

In the last 20 years, numerous clinical trials have been implemented to test new anti-amyloid and tau-targeting drugs for the treatment of AD. Unfortunately, despite the enormous efforts, both scientific and economic [49], only in 2021 did the US Food and Drug Administration (FDA) approve, with some concern, aducanumab, a human IgG1 anti-Aβ monoclonal antibody selective for Aβ aggregates, as the first disease-modifying treatment for AD [50].

In the light of increasing evidence supporting an involvement of PARylation in normal neuronal functions as well as in neurodegeneration and neuropathology [12,51], we reasoned that PARP-1 could represent a new interesting pharmacological target in AD, and PARP-1 inhibitors could become the objects of a drug-repurposing evaluation to identify a new beneficial treatment for AD.

Animal models are a powerful tool to investigate the in vivo pathophysiological processes that cause neurodegenerative diseases. *Drosophila melanogaster* is an excellent choice for modelling the biochemical, genetic, and behavioral aspects of AD because it contains a fully functional nervous system with an architecture that separates specialized functions such as vision, olfaction, learning and memory similarly to mammalian nervous systems [52,53,54]. Moreover, the majority of pathological features of AD can be recapitulated in transgenic fly models, including neurotoxicity, nuclear inclusion formation, progressive neurodegeneration, behavioral abnormalities and early death [55,56,57,58]. In addition, biochemical pathways that are affected in AD, such as detoxification, protein clearance and stress responses, are conserved between flies and humans (see [59] for a review). In *Drosophila*, the PARP family includes only two members, a nuclear PARP-1 that is responsible for the majority of PAR synthesis [60,61], and PARP-5 (tankyrase) located in the cytoplasm [62,63]. For all the above reasons, *Drosophila* is an excellent system for dissecting the molecular pathways involved in AD and screening disease-modifying drugs.

In this study, two different transgenic *Drosophila* AD models were employed to evaluate the potential therapeutic effects of PARP inhibitors: olaparib [64], already approved for the treatment of several cancer types (for a review see [65]) and MC2050, a new inhibitor, previously developed by our team, that has been shown to have neuroprotective effects in cellular models of Aβ peptides-induced neurotoxicity [35,66]. We assessed the contribution of PARP inhibition to the molecular mechanisms involved in AD progression by focusing on several disease-specific features, including AD-like phenotypes such as locomotor dysfunction, reduced lifespan and epigenetic dysregulation of transposable elements activity.

## 2. Materials and Methods

### 2.1. Fly Stocks

All transgenic fly stocks used in this study were obtained from the Bloomington *Drosophila* stock center (BDSC, Indiana University, Bloomington, IN, United States, Available online: http://flystocks.bio.indiana.edu/ accessed on 15 March 2022) and are listed below: UAS-Aβ42 (w^1118^; P{UAS-APP.Aβ42.B}m26a, #33769); UAS-APP, BACE1 (w^1118^; P{UAS-BACE1.Exel}7b, P{UAS-APP.695.Exel}1/TM6B, Tb^1^, #33797); elav-Gal4 (P{GawB}elav^C155^ #458); elav-Gal4, UASmCD8::GFP (P{GawB}elav^C155^, P{UAS-mCD8::GFP.L}Ptp4E^LL4^, P{hsFLP}1, w* #5146). PARP-1 RNAi stocks (y^1^ sc* v^1^ sev^21^; P{TRiP.HMC04658}attP40, #57265, and y^1^ sc* v^1^ sev^21^; P{TRiP.HMS01233}attP2, #34888). A detailed description of all the lines is given at FlyBase (Available online: http://flybase.org/ accessed on 15 March 2022). Balancers used to balance inserts on the second and third chromosomes, respectively, have been kept in our laboratory for many years.

To generate *elav-Gal4* > *Aβ42*; *PARP-1^RNAi^* flies, virgin females carrying the pan-neuronal *elav-Gal4* driver on the X chromosome were crossed to males carrying both *UAS-Aβ42* and *UAS-PARP-1^RNAi (^*^HMC04658)^ transgenic constructs on the second and third chromosome, respectively. To obtain *elav-Gal4* > *PARP-1^RNAi^*; *APP*, *BACE1* flies, virgin females *elav-Gal4* were crossed to males carrying both *UAS-PARP-1^RNA1 (HMS01233)^* and *UAS-APP, BACE1* on the second and third chromosome, respectively.

All flies were raised at 25 °C on a standard cornmeal-sucrose-yeast-agar medium. All crosses were performed at 25 °C except for RNAi crosses that were set up at 29 °C to optimize the PARP-1 knockdown. The Ore-R stock and balancer stocks used here have been kept in our laboratory for many years under standard conditions.

### 2.2. Pharmacological Inhibition of PARP-1

Olaparib (AZD2281, Selleckchem, Radnor, PA, USA) and MC2050 (2-[2-(4-(2-pyridyl)-1-piperazinyl) ethylsulfanyl]-3H-quinazolin-4-one) dihydrochloride (kindly provided by Rotili lab, Sapienza University, Roma, Italy) were used as specific inhibitors of PARP-1 [66,67]. MC2050 was directly dissolved in water at an initial concentration of 50 mM and then was added to the standard medium to a final concentration of 50 µM or 100 µM. Olaparib was dissolved at a concentration of 30 mM in DMSO and then diluted to a final concentration of 25 μM in the medium.

### 2.3. Climbing Assay

A group of 10 male or female flies was tested for climbing ability; the number of flies per group that climbed above the 8 cm mark by 15 s after the tap was measured, recorded as the percentage success rate. Ten trials were performed for each group and *n* ≥ 100 flies were assayed for each genotype. Experiments were performed during daylight to minimize potential effects of circadian oscillation. All average data are presented as mean ± SEM.

### 2.4. Lifespan Assay

Control and experimental flies were reared at 25 °C on standard sugar-yeast medium supplemented with inhibitors. To estimate the longevity of each experimental group, 150–200 flies were collected within 24 h after eclosion. Flies were transferred to a fresh medium three times a week and dead flies were counted daily. The survival rate was calculated as the percentage of total surviving flies. For each experiment, at least two biological replicates were pooled. The survival rate was estimated using the Kaplan–Meier procedure and plotted as survival curves. Mean, median, minimum and maximum lifespan and the age of 90% mortality were calculated.

### 2.5. NAD^+^ Measurement

NAD^+^ was quantified in *Drosophila* brains using NAD^+^/NADH Quantification kit (Merck KGaA, Darmstadt, Germany). Briefly, 10 total brains obtained from *elav-Gal4* > *Aβ42* treated with or without PARP-1 inhibitors were lysed in NAD^+^/NADH extraction buffer. *Elav-Gal4/+* brains were extracted in the same buffer and it was regarded as control. The assay was carried out according to the manufacturer’s instructions and the results were expressed as NAD^+^ levels (pmol/10 brains). The experiment was performed in at least three biological replicates.

### 2.6. Western and Slot Blot

Total proteins were extracted from adult heads/brains in SDS loading buffer (60 mM Tris-HCl pH 6.8, 10% glycerol, 2% SDS, 10 mM dithiothreitol and 0.1% bromophenoblue). For the Slot blot, brain samples were homogenized in an SDS sample buffer solution without bromophenol blue and containing 3% SDS. The protein extracts were applied on a nitrocellulose membrane according to Bio-Dot SF protocol application (Bio-Rad Laboratories, Hercules, CA, USA) and air-dried. For the Western blot, proteins resolved by 4–20% SDS-PAGE were transferred onto PVDF membrane with a TransBlot Turbo semi-dry blotting apparatus (Bio-Rad Laboratories). Membranes were incubated with 5% BSA solution and probed with the following antibodies: anti-beta amyloid (1–16) antibody (6E10 clone 1:500, Sigma), H3 pan-acetyl (1:1000, Millipore, Burlington, MA, USA), H3K27me3 (1:1000, Cell Signaling, Danvers, MA, USA), H3K9me3 (1:1000, Abcam, Cambridge, UK), PARP-1 (1:1000, kindly provided by Gagné-Poirier lab., Laval University, Canada), α-PAR (1:1000, EnzoLife, Farmingdale, NY, USA, 10H mAb), tubulin (1:5000, Sigma), actin (1:5000, Millipore-Merck) and histone H3 (1:5000, Abcam). The secondary antibodies were HRP-conjugated anti-mouse and anti-rabbit used at 1:5000 dilution (Bio-Rad). The target proteins were visualized by the ECL system (Bio-Rad) and the densitometric analyses were performed with ImageLab v6.1 software (Bio-Rad) and normalized to tubulin, actin and histone H3. Three biological replicates were performed with three technical replicates.

### 2.7. RNA Isolation and qRT-PCR Analysis

Total RNA samples were purified from adult heads using Qiazol reagent (Qiagen, Hilden, Germany) according to the manufacturer’s instructions. RNA concentration and purity were determined using a NanoDrop 1000 spectrophotometer (Thermo Scientific, Waltham, MA, USA). In total, 5 µg of total RNA was reverse-transcribed using oligo dT and SuperScript™ Reverse Transcriptase III (Invitrogen, Waltham, MA, USA) according to the manufacturer’s protocol. The qPCR reactions were carried out with QuantiFast SYBR Green PCR Kit (Qiagen) according to the manufacturer’s protocol. Relative abundance of the different transcripts was determined using the 2^−ΔΔCT^ method [68] using *rp49* transcript as a control. qRT-PCR experiments were performed in three independent biological replicates, each with three technical replicates. The primers used were:

parp-1 F: 5′-CCTTTGTGGCATCATTTGGA-3′

parp-1 R: 5′-ACGCAAACCAGCCAAGCT-3′

opus F: 5′-CGAGGAGTGGGGAGAGATTG-3′

opus R: 5′-TGCGAAAATCTGCCTGAACC-3′

roo F: 5′-CGTCTGCAATGTACTGGCTCT-3′

roo R: 5′-CGGCACTCCACTAACTTCTCC-3′

springer F: 5′-CCATAACACCAGGGGCA-3′

springer R: 5′-CGAGTGCTGGTCTGTCA-3′

copia F: 5′-TGGAGGTTGTGCCTCCACTT-3′

copia R: 5′-CAATACCACGCTTAGTGGCATAAA-3′

Aurora F: 5′-GAAGGAACTGAGCGTGTTCCA-3′

Aurora R: 5′-CGTCTACCGCAACTAATGCAAA-3′

Aβ42 F: 5′-TTCCGACATGACTCAGGATATGA-3′

Aβ42 R: 5′-CCAATGATTGCACCTTTGTTTG-3′

rp49 F: 5′-GCGCACCAAGCACTTCATC-3′

rp49 R: 5′-TTGGGCTTGCGCCATT-3′.

### 2.8. Drosophila Adult Brain Immunofluorescence

Immunofluorescence staining of adult brains was performed according to Wu and Luo [69]. Briefly, adult brains were dissected in PBS, fixed with 4% paraformaldehyde in PBS for 30 min at room temperature, washed three times for 10 min in PBT (PBS with 0.1% Triton X-100) and blocked in PBT-NGS (5% normal goat serum in PBT) for 30 min at 4 °C. Brains were then incubated overnight at 4 °C with primary monoclonal anti-β-amyloid (1–16) antibody (6E10 clone) diluted 1:100 in PBT-NGS. After 3 washes for 10 min in PBT, brains were incubated with secondary goat anti-mouse antibodies conjugated with AlexaFluor 568 fluorophore (1:300 in PBT) and stained with TOTO-3 iodide (1 μM) to visualize DNA. Confocal images were acquired on a Leica DMIRE (Leica Microsystems, Heidelberg, Germany) and a Zeiss LSM 780 (Zeiss, Berlin, Germany) microscope. Images were analyzed and further processed using Zen Software (ZEN 2009 Light Edition) and Adobe Photoshop CS6.

To count β-amyloid positive puncta, 6 brains from *elav-Gal4* > *Aβ42* flies, treated or not with PARP-1 inhibitors, were fully optically sectioned by confocal microscopy. The number of β-amyloid puncta in each brain section was counted using ImageJ/Fiji plugin to count spots (Spot Counter Plug-in version 0.14) and averaged between each brain. Statistical significance was determined using an unpaired Student’s *t*-test.

### 2.9. Statistical Analysis

Statistical analyses were performed using GraphPad Prism version 6.00 (GraphPad Software, La Jolla, CA, USA). Data were expressed as mean values ± SEM from at least three biological independent experiments. For all statistics, a *p* value ≤ 0.05 was considered statistically significant. qRT-PCR analysis was performed by one- or two-way ANOVA test (followed by Tukey’s multiple comparison test). Western blots were analyzed by unpaired t-test or two-way ANOVA test (followed by Tukey’s post hoc multiple comparison test). Lifespan data were analyzed by log rank test with Bonferroni adjustment. Climbing data were compared by one- or two-way ANOVA followed by Tukey multiple comparison tests. For NAD^+^ quantification levels, one-way ANOVA followed by Tukey’s multiple comparison test was applied. Statistical comparison of 6E10 positive puncta was performed by unpaired t-test.

## 3. Results

### 3.1. Pharmacological Inhibition or RNAi-Mediated Genetic Knockdown of PARP-1 Ameliorates Aβ42-Induced Locomotor Defects in Aβ42 Model of AD

The impairment of locomotor coordination is an important hallmark of neurotoxicity in different neurodegenerative disorders, including AD [70]. All transgenic *Drosophila* models of AD show a severe and progressive age-dependent loss of climbing ability [71,72].

In order to study the neuroprotective and therapeutic potential of PARP-1 functional inhibition in vivo, we evaluated the climbing ability of transgenic AD flies expressing the human Aβ42 peptide under the control of the heterologous upstream activation sequence (UAS) yeast sequences. The neuron-specific expression of the transgenic construct was achieved through the transcriptional activator Gal-4 under the control of the pan-neuronal elav promoter. The neuronal specific expression of Aβ42 in AD flies can recapitulate all the pathophysiological characteristics of AD, including impaired locomotor capacity [72,73,74].

AD flies expressing Aβ42 were allowed to feed on standard sugar-yeast medium supplemented with olaparib (25 µM) or MC2050 (100 µM) for the entire developmental period and were then assayed for climbing activity at 10–12 days post-eclosion. This life-period corresponds to the time window in which the phenotypic profile related to neuronal cell death and locomotor deficits is already detectable even if AD flies are not in the exponential decline period of their lifespan.

It is noteworthy that our choice of PARP-1 inhibitors was based on their chemical properties. In particular, the water solubility of MC2050 has allowed us to use it without any previous dissolution in DMSO, as requested by olaparib. Moreover, MC2050 has been observed in the plasma and the brain tissue of CD1 mice (data not shown).

**Figure 1:**
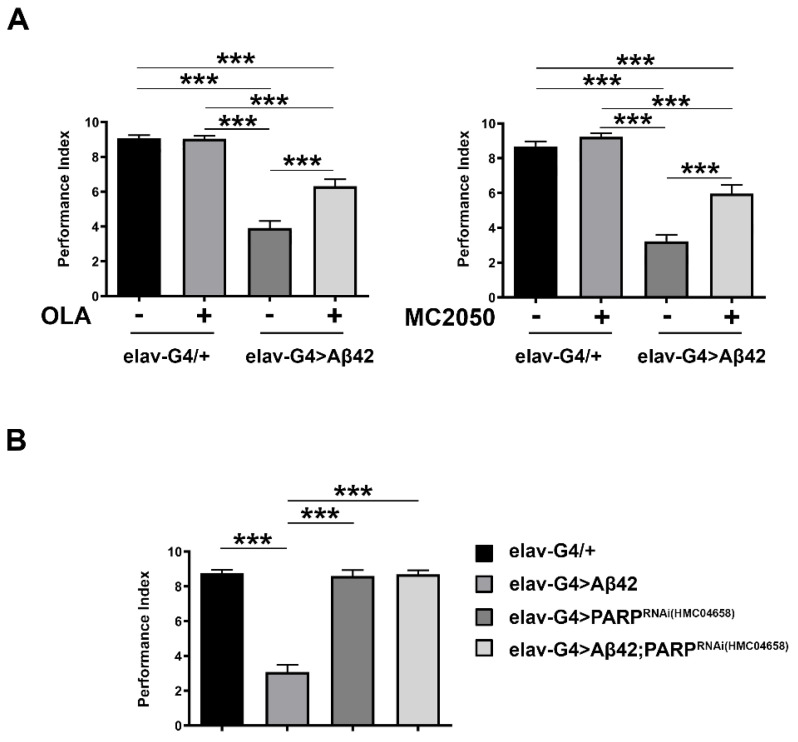
(**A**) Climbing test on *elav-Gal4* > *Aβ42* and *elav-Gal4/+* genotypes, in the presence and absence of olaparib and MC2050 PARP-1 inhibitors. Statistical significance was determined using the two-way ANOVA test followed by the Tukey test for multiple comparisons (*** *p* < 0.001). (**B**) Climbing test on AD Aβ42 flies with neuron-specific depletion of PARP-1 by RNAi. Statistical significance was determined using the one-way ANOVA test followed by the Tukey test for multiple comparisons (*** *p* <0.001).

The results obtained showed that PARP-1 inhibition significantly ameliorates the impaired climbing performance of Aβ42 flies (*elav-Gal4* > *Aβ42*) when compared to the vehicle-treated control flies (Figure 1A), but it has no effect on motor performance of control flies (*elav-Gal4/+*).

To confirm the data obtained with PARP-1 inhibitors and rule out their non-specific effects, we tested the potential neuroprotective effect of RNAi-mediated genetic knockdown of PARP-1. We generated a transgenic line expressing both Aβ42 peptide and short inverted repeat interfering PARP-1 (HMC04658), under the control of the pan-neuronal driver elav-Gal4; the transgenic strain *elav-Gal4* > *Aβ42*; *PARP-1^RNAi (HMC04658)^* allowed us to analyze the climbing activity of AD flies in a genetic background depleted of PARP-1. Quantitative RT-PCR (qRT-PCR) was used to verify the effective depletion of PARP-1 following in vivo RNAi (Supplementary Figure S1A).

Consistently with the effects observed for PARP-1 inhibitors, quantitative analysis of climbing defects following the combined pan-neuronal expression of Aβ42 and RNAi-based PARP-1 knockdown showed a significant recovery of locomotor activity in *elav-Gal4* > *Aβ42*; *PARP-1^RNAi^* flies relative to *elav-Gal4* > *Aβ42* flies. Notably, the analysis of climbing ability did not reveal consistent defects between *elav-Gal4* > *PARP-1^RNAi^* and healthy controls (*elav-Gal4/+*) (Figure 1B).

To account for a possible titration of available Gal4 across multiple UAS-driven targets and subsequent reduction in the expression of the pathogenic construct *A*β*42,* we repeated climbing experiments by adding a second unrelated UAS transgene (UAS-mCD8::GFP) in the *elav-Gal4* > *Aβ42* genetic background. As shown in Supplementary Figure S2A, this additional control did not change the experimental outcome, confirming that the results shown in Figure 1A were not due to a dilution of Gal4 protein in the presence of two UAS transgenes.

These results agree with and strengthen the data obtained from the pharmacological inhibition of PARP-1 underlining the importance of PARP-1 in neurodegeneration.

### 3.2. PARP-1 Inhibitors Improve NAD^+^ Content

**Figure 2:**
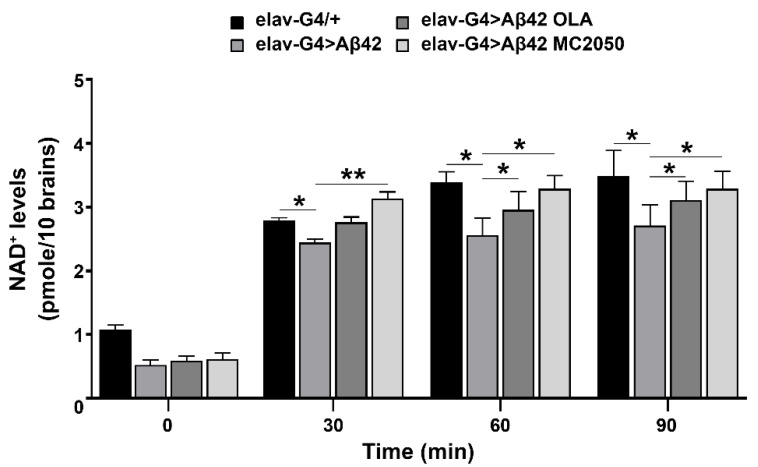
PARP-1 induces NAD^+^ depletion. NAD^+^ levels were determined by the use of a dedicated colorimetric kit from Merck-Sigma-Aldrich, following manufacturer’s instructions. *Elav-Gal4* > *Aβ42* flies were treated as described in the Material and Methods section. *Elav-Gal4/+* was considered as control. Data represented mean (±SEM) of three independent experiments. Statistical analysis was performed by one-way ANOVA and Turkey post hoc test (* *p* < 0.05, ** *p* < 0.01).

Several authors pointed out that increased expression and activity of PARP-1 drives NAD^+^ depletion [10,75]. To assess whether PARP inhibition leads to NAD^+^ increase, thus contributing to climbing recovery, we performed NAD^+^ determination. As shown in Figure 2, inhibitor treatments significantly prevent NAD^+^ consumption observed in *elav-Gal4 > Aβ42* flies.

### 3.3. PARP-1 Inhibitors Impair PARylation without Affecting Aβ42 or PARP-1 Protein Expression

**Figure 3:**
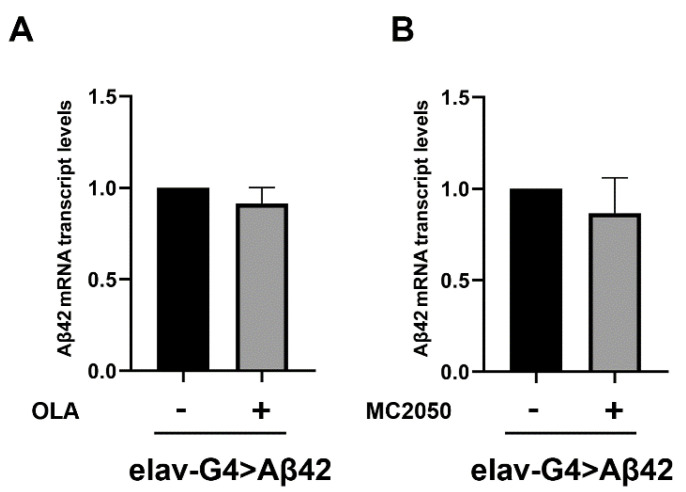
(**A**,**B**) qRT-PCR analysis assessing induction levels of the Aβ42 transgenic construct by elav-Gal4. cDNA was prepared from total RNA purified from AD (*elav-Gal4 > Aβ42*) head tissues treated (or not) with PARP-1 inhibitors. The constitutive *rp49* was examined as an endogenous control. The data are expressed as “fold enrichment” with respect to *elav-Gal4/+* and represent the mean ± SEM of three independent experiments. Statistical analysis, determined by unpaired t-test, showed no significant difference between means.

To rule out a putative effect of PARP-1 inhibitors on Aβ42 transcription, we performed qRT-PCR on RNA samples purified from head tissues of the same flies tested for climbing and the results showed no significant changes in the expression profile of the pathogenic construct Aβ42 following PARP-1 inhibition (Figure 3).

These results strongly suggest that PARP-1 pharmacological inhibition could exert, in vivo, a neuroprotective effect in AD.

**Figure 4:**
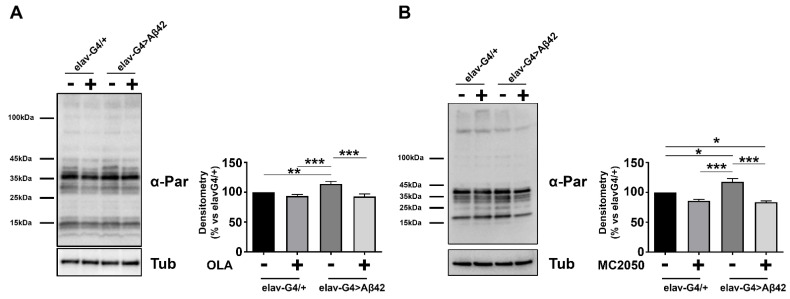
Protein extracts obtained from *elav-Gal4/+* and *elav-Gal4* > *Aβ42* head tissues, at 10–12 days of age, were analyzed by SDS-PAGE, transferred onto PVDF membrane, and probed with antibodies against α-PAR. Densitometry analysis was performed with ImageLab software; total PARylated protein lane samples were normalized with Tubulin. Data are expressed as a percentage of the *elav-Gal4/+* control values and represent the mean ± SEM of at least three independent experiments. Statistical significance was determined using the two-way ANOVA test followed by the Tukey test for multiple comparisons (* *p* < 0.05, ** *p* < 0.01, *** *p* < 0.001).

In order to quantify the efficiency of olaparib and MC2050 in compromising PARP-1 activity, we analyzed the pattern of PARylated cellular proteins after each pharmacological inhibitor treatment (Figure 4). Whole cell lysate from AD (*elav-Gal4* > *Aβ42*) and control (*elav-Gal4/+*) head tissues, treated or untreated (vehicle only), were analyzed by Western blotting using an anti-PAR antibody. As shown in Figure 4, densitometric analysis of PARylation revealed a significant increase in PARylation levels in *elav-Gal4* > *Aβ42* samples relative to control *elav-Gal4/+* samples, similar to previous results obtained in mouse and human models, an observation that suggests that the activation of PARP-1 is an important early event in the pathogenesis of AD. As expected, PARylation levels of AD flies treated with PARP-1 inhibitors lie in a range comparable to that of the vehicle-treated control (Figure 4).

**Figure 5:**
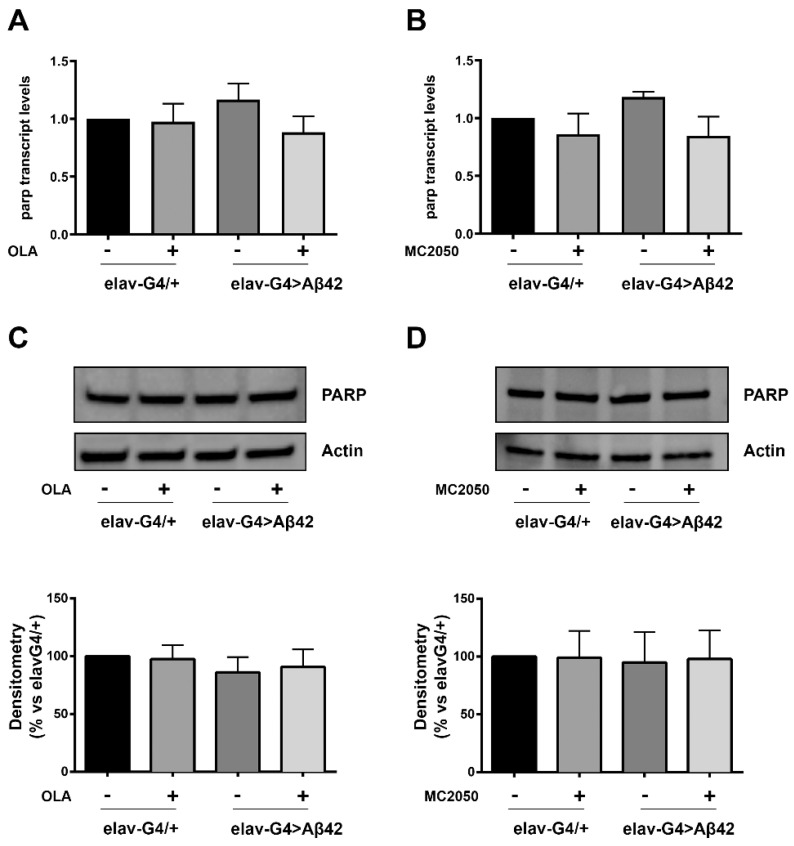
(**A**,**B**) qRT-PCR analysis assessing induction levels of PARP-1 transcript following olaparib (**A**) or MC2050 (**B**) treatments. cDNA was prepared from total RNA purified from AD (*elav-Gal4* > *Aβ42*) and control (*elav-Gal4/+*) head tissues, treated (or not) with PARP-1 inhibitors. The constitutive *rp49* was examined as an endogenous control. The data are expressed as “fold enrichment” with respect to *elav-Gal4/+* and represent the mean ± SEM of three independent experiments. Statistical analysis, determined by unpaired t-test, showed no significant difference between means. (**C**,**D**) Western blot analysis of head protein extracts from *elav-Gal4/+* and *elav-Gal4* > *Aβ42* flies, treated or not with olaparib (**C**) or MC2050 (**D**), probed with PARP-1 specific antibody. Densitometry analysis was performed using ImageLab software and normalized to Actin. The data are expressed as a percentage of the *elavG4/+* control values and represent the mean ± SEM of at least three independent experiments. Two-way ANOVA followed by the Tukey test for multiple comparisons showed no significant differences among the means of the different groups.

In addition, we quantified *parp-1* mRNA levels by quantitative RT-PCR assay (qRT-PCR) in head tissues from *elav-Gal4* > *Aβ42* and *elav-Gal4/+* flies and PARP-1 protein levels by Western blot using anti-PARP-1 antibodies. Results showed no significant changes in *parp-1* mRNA and PARP-1 protein levels between *elav-Gal4* > *Aβ42* and *elav-Gal4/+* samples (Figure 5). No changes in PARP-1 expression profiles following olaparib or MC2050 treatments were detected, thus suggesting that in our AD model, the enzymatic activity of PARP-1 following inhibitor treatments is not directly correlated with its transcriptional regulation.

### 3.4. Olaparib and MC2050 Inhibits Aggregation of Aβ42 Peptides in AD Adult Brains

Aβ oligomers and plaques are specific neuropathological hallmarks of AD [76,77]. Aβ is produced through the sequential proteolytic processing of APP [78] and its progressive accumulation in brain senile plaques is proposed to be an early toxic event leading to neuronal degeneration and death [79]. Therefore, Aβ42 is predicted to be the most potentially efficient target of drug therapies [80]. In a previous work, Iijima and collaborators reported that flies expressing human Aβ42 peptides raised at 25 °C exhibit amyloid-containing puncta in their brain compared to controls [81].

In order to investigate whether PARP-1 inhibitors can interfere in vivo with Aβ42 aggregation, we performed whole-mount immunostaining on *elav-Gal4* > *Aβ42* brains with 6E10 antibody which is directed against amino acids 6–10 of amyloid-β (Aβ).

**Figure 6:**
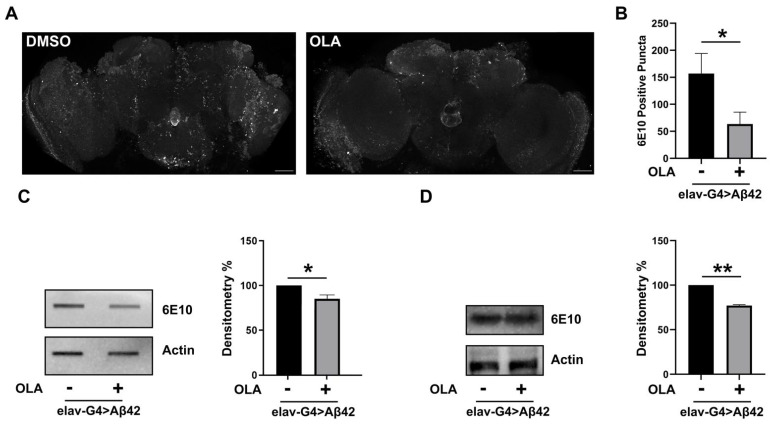
(**A**) Confocal images of representative fly brains from 15-day-old *elav-Gal4* > *Aβ42* flies raised on standard medium supplemented with DMSO or olaparib showing immunoreactivity to anti-amyloid-β antibody (6E10). Scale bar indicates 50 μm. (**B**) Histogram depicts the quantification of 6E10-positive puncta. *n* = 6 adult brains. Error bars represent SEM. (* *p* < 0.05). (**C**,**D**) Slot and Western blot analysis of brain protein extracts from *elav-Gal4* > *Aβ42* flies probed with Aβ42 specific antibody (6E10) (left panel). Quantification (right panel) was made using Actin for normalization and results are expressed as percentage relative to levels measured in vehicle-treated *elav-Gal4* > *Aβ42* flies. Statistical significance was determined by unpaired *t*-test (* *p* < 0.05, ** *p* < 0.01).

In the brains of transgenic Aβ42 flies, pan-neuronal expression of secreted Aβ42 (using the elav-Gal4 driver) promotes the formation of abundant amyloid-β-positive toxic aggregates, the number and intensity of which were both reduced following olaparib treatment (Figure 6A,B). We obtained similar effects with the MC2050 treatment (data not shown). Results obtained by whole-mount immunofluorescence on *elav-Gal4* > *Aβ42* adult brains were confirmed by Slot and Western blot analysis (Figure 5C,D). Although the mechanistic details remain unclear and need further study, these observations point to an important and unexplored role of PARP-1 in Aβ42 toxic oligomers self-aggregation.

### 3.5. Effects of PARP-1 Inhibitors on the Transposable Elements’ Expression

It is well known that transposable elements (TE) uncontrolled activation represents a common feature of several neurodevelopmental and neurodegenerative disorders, making them an interesting common denominator, which opens up possibilities for alternative diagnostic and treatment strategies [82,83,84]. Rett syndrome was the first neurodevelopmental disorder in which an increase in somatic L1 retrotransposon insertions was discovered [85]. Pathological TE activation has also been observed in ataxia telangiectasia [86], macular degeneration [87], prion disease [88], schizophrenia [89], Parkinson’s disease [90], amyotrophic lateral sclerosis (ALS) [91,92,93] and Huntington’s disease (HD) [94]. It is noteworthy that integrated studies on human postmortem brains from patients with AD and tau-expressing *Drosophila* models demonstrated that, also in AD, aberrant activation and mobilization of TEs substantially contribute to neurodegeneration [95,96,97]. To verify whether expression profiles of TEs could be dysregulated in the Aβ42 model and to understand whether PARP-1 inhibition could modulate Aβ42-induced TE activation, we performed qRT-PCR experiments on RNA samples purified from *elav-Gal4* > *Aβ42* and *elav-Gal4/+* head tissues, in the presence or absence of PARP-1 inhibitors.

**Figure 7:**
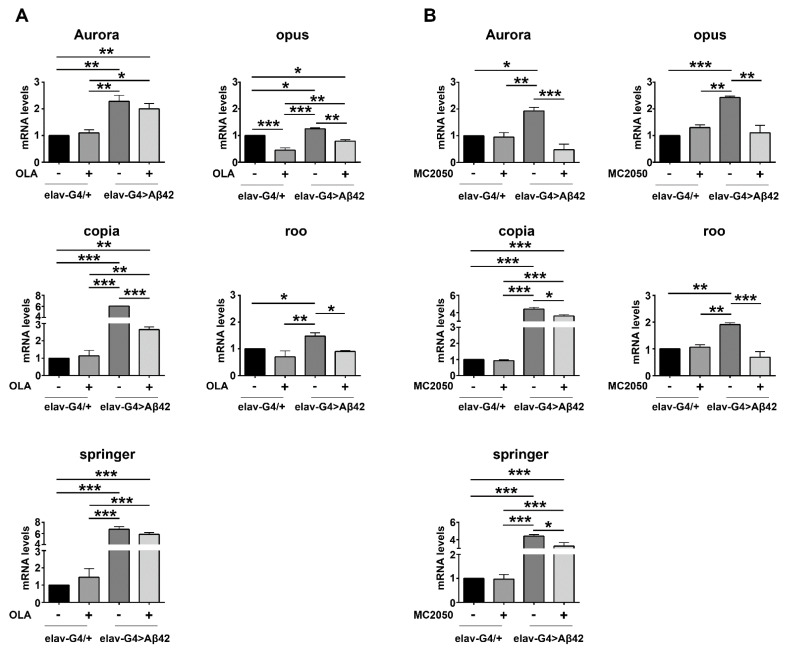
qRT-PCR analysis of transposable element expression in head tissues of flies expressing the pathogenic construct Aβ42 in neurons (*elav-Gal4* > *Aβ42*) treated or not with PARP-1 inhibitors olaparib (**A**) or MC2050 (**B**) relative to untreated controls (*elav-Gal4/+*); heads were analyzed at 10–12 days post-eclosion; transcript levels were normalized to *rp49* and displayed as fold change relative to untreated control flies (*elav-Gal4/+*). Statistical significance was determined using the two-way ANOVA test followed by the Tukey test for multiple comparisons (* *p* < 0.05, ** *p* < 0.01, *** *p* < 0.001).

As shown in Figure 7, we found that expression levels of Aurora, opus, copia, roo and springer were significantly increased in *elav-Gal4* > *Aβ42* AD flies compared to *elav-Gal4/+* control flies, indicating that TE activation actually represents an intrinsic feature of AD. Notably, PARP-1 inhibition had no effect on TE expression (except for opus) in control flies, but strongly suppressed Aβ42-induced TE dysregulation in *elav-Gal4* > *Aβ42* AD flies, indicating again that the functional correlation between PARP-1 activity and TE expression could be crucial in determining the AD pathological phenotype.

### 3.6. PARP-1 Inhibition Restores Histone Modifications in the Aβ42 Model

The involvement of PARP-1 in controlling epigenetic modifications and chromatin architecture in a context-dependent manner makes PARylation an important player in the epigenetic control of transcriptional activity of genes potentially implicated in the pathogenesis of AD. Although the precise molecular mechanisms underlying the contribution of PARP-1 to chromatin organization and transcriptional regulation are sophisticated and remain not completely defined, it is clear that PARP-1 is able to regulate the complex interplay between histone acetylation and methylation by modulating the activity of histone and chromatin-modifying enzymes [98,99,100,101].

**Figure 8:**
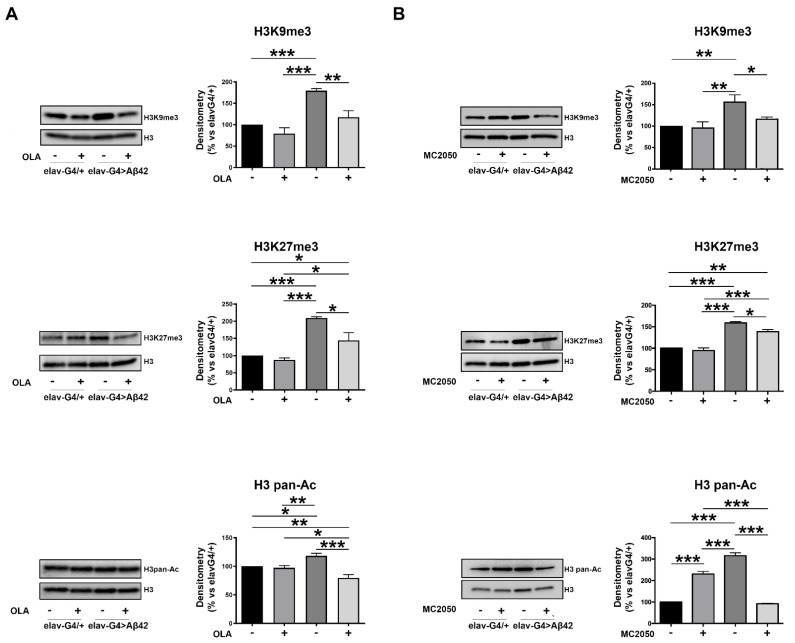
Total protein extracts obtained from head tissues of *elav-Gal4/+* and *elav-Gal4* > *Aβ42* flies, treated or not with PARP-1 inhibitors olaparib (**A**) or MC2050 (**B**), were resolved by SDS-PAGE, transferred onto PDVF membrane and probed with antibodies against histone H3 trimethylated in lysine 9 (H3K9me3), trimethylated in lysine 27 (H3K27me3) and histone H3 pan-acetylated (H3 pan-Ac). Densitometry analysis was performed using ImageLab software and normalized to H3. The data are expressed as a percentage of the *elav-Gal4/+* control values and represent the mean ± SEM of at least three independent experiments. Statistical significance was determined using the two-way ANOVA test followed by the Tukey test for multiple comparisons. (* *p* < 0.05; ** *p* < 0.01; *** *p* < 0.001.)

In order to investigate whether histone acetylation and/or methylation were deregulated in our AD Aβ42 model and to verify whether PARP-1 inhibition with olaparib and MC2050 may affect histone acetylation and/or methylation levels, we performed Western blotting experiments of epigenetic markers from head tissue of *elav-Gal4* > *Aβ42* and *elav-Gal4/+* control flies. As shown in Figure 8, we found a significant increase of H3 pan-acetylated, H3K27 trimethylated and H3K9 trimethylated in *elav-Gal4* > *Aβ42* relative to the control *elav-Gal4/+*. Notably, the treatments with olaparib on *elav-Gal4* > *Aβ42* flies restore H3 pan-acetylation and both H3K9 and H3K27 trimethylation, whilst it seems to have no effect on these histone modifications in the *elav-Gal4/+* genotype. Although related to a limited number of histone modifications, these results demonstrated that PARP-1 inhibition can restore the histone modification landscape altered in AD.

### 3.7. RNAi-Mediated Gene Silencing of PARP-1 Rescues Motor Dysfunction and Improves the Life Expectancy of a Transgenic Model Expressing Human APP and BACE1 (APP/BACE1 Model)

To assess the neuroprotective effect of PARP-1 knockdown on Aβ-induced neurotoxicity, we extended our analysis to a different transgenic AD model expressing the human amyloid precursor (APP695) protein and the β-secretase enzyme BACE1 within the central nervous system. APP protein is cleaved by transgenic human BACE1 and then by endogenous *Drosophila* γ-secretase to generate Aβ peptides in the brain [102]. This *Drosophila* model is another powerful tool for AD research as the files exhibit several clinical AD neuropathology and symptomology for both familial and sporadic AD, including Aβ42 aggregation, synaptic abnormalities at the neuromuscular junction and behavioral and memory defects [71,102,103].

To investigate whether PARP-1^RNAi^ may ameliorate APP/BACE-induced motor dysfunction, we generated transgenic flies overexpressing the PARP-1^RNAi^ construct (HMS01233) in the APP/BACE1 model and quantified the climbing ability for each genotype combination after checking by qRT-PCR the effective genetic depletion of PARP-1 following in vivo RNAi (Supplementary Figure S1B).

**Figure 9:**
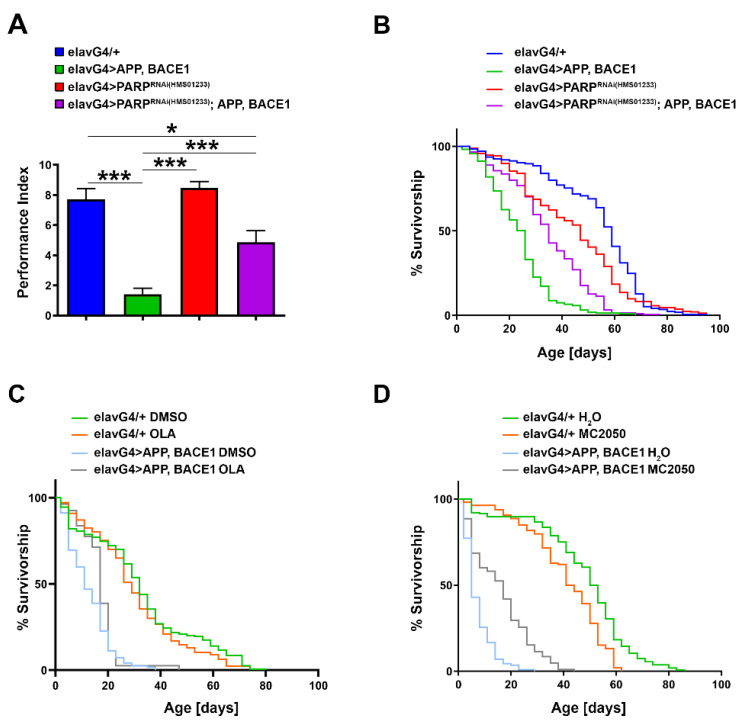
(**A**) Climbing test on AD APP/BACE1 flies with neuron-specific depletion of PARP-1 by RNAi. Statistical significance was determined using the one-way ANOVA test followed by the Tukey test for multiple comparisons (* *p* < 0.01; *** *p* < 0.001). (**B**) Survival curves of AD APP/BACE1 flies with neuron-specific depletion of PARP-1 by RNAi and related controls. The log-rank (Mantel–Cox) test with Bonferroni correction for multiple comparisons indicates a significant difference between all survival curves (*** *p* < 0.001). (**C**) Survival curves of AD APP/BACE1 flies treated with 25 µM olaparib and related controls. The log-rank (Mantel–Cox) test with Bonferroni correction for multiple comparisons indicates a significant difference between all survival curves (* *p* < 0.01) except for *elav-Gal4/+* DMSO vs. *elav-Gal4/+* OLA. (**D**) Survival curves of AD APP/BACE1 flies treated with 50 µM MC2050 and related controls. The log-rank (Mantel–Cox) test with Bonferroni correction for multiple comparisons indicates a significant difference between all survival curves (*** *p* < 0.001).

Consistent with the data collected in the Aβ42 model, we observed that *elav-Gal4* > *APP*, *BACE1* flies have reduced climbing activities, indicating that APP and BACE1 compromised CNS functions. Remarkably, knockdown of PARP-1 completely rescued the APP/BACE-induced motor impairment (Figure 9A). To rule out a possible titration of available Gal4 in the presence of multiple UAS transgenes, we repeated climbing experiments by adding the unrelated UAS-mCD8::GFP transgene in the *elav-Gal4* > *APP*, *BACE1* genetic background. As shown in Supplementary Figure S2B, this additional control confirms the results presented in Figure 9A.

It was previously reported that pan-neuronal co-expression of APP and BACE-1 strongly reduced the lifespan of the adult flies [71]. To test whether PARP-1 knockdown was able to rescue the reduced lifespan of *elav-Gal4* > *APP, BACE1* flies, we performed lifespan assays using the same genotypes tested for climbing experiments. The log-rank analysis of survival curves showed that median survival of *elav-Gal4* > *APP, BACE1* flies was markedly decreased (55.9%) compared with *elav-Gal4/+* control flies (the median survival was 26 days for *elav-Gal4* > *APP*, *BACE1* flies vs. 59 days for *elav-Gal4/+*). Depletion of PARP-1 significantly reduced the lifespan of control flies by 20.3% (the median survival was 47 days for *elav-Gal4* > *PARP-1^RNAi^* flies vs. 59 days for *elav-Gal4/+*), but significantly increased the median lifespan of *elav-Gal4* > *APP*, *BACE1* by 34.6% (the median survival was 26 days for *elav-Gal4* > *APP*, *BACE1* flies vs. 35 days for *elav-Gal4* > *PARP-1^RNAi^*; *APP*, *BACE1*) (Figure 9B).

To verify whether PARP-1 inhibition could have relevant systemic effects in rescuing reduced lifespan of *elav-Gal4* > *APP*, *BACE1* flies, we performed lifespan experiments by feeding AD (*elav-Gal4* > *APP*, *BACE1*) and control (*elav-Gal4/+*) flies on a standard cornmeal-sucrose-yeast-agar medium supplemented with 25 μM olaparib or 50 μM MC2050 over the entire developmental period. For MC2050, we used a final concentration of 50 μM since the 100 μM concentration, even if it is very effective for short-term exposures, appeared to be toxic over a long period of time. The log-rank analysis of survival curves showed that both olaparib and MC2050 treatments significantly prolonged the median survival of AD flies by 32 and 300% respectively, as compared to vehicle-treated flies (the median survival was 18.5 days for olaparib-treated AD flies vs. 14 days for DMSO-treated AD flies and 20 days for MC2050-treated flies vs. 5 days for untreated AD flies) (Figure 9C,D).

Unexpectedly, MC2050 treatment resulted in a mild toxic effect on the control *elav-Gal4/+* genotype: 50% of MC2050-treated control flies died at 42.5 days, while 50% of the untreated group died at 51.5 days (Figure 9D). The observed toxic effect could be explained by the important physiological roles played by PARP-1 in cellular physiology and in genomic stability (refer to [104] for a review).

## 4. Discussion

Despite growing public attention and funding directed into dementia research, the fundamental cause of AD remains elusive, providing no treatment or intervention strategy against the molecular events that constitute its pathophysiology. In the last decade, a growing body of experimental evidence suggests that PARP-1 is activated in neurodegenerative diseases, neurodevelopmental disorders and aging, leading to neuroinflammation, autophagy dysregulation and mitochondrial dysfunction [105,106,107,108]. Kam et al. [109] reported that in Parkinson’s disease (PD), pathologic a-synuclein accumulation induces PARP-1 hyperactivation and that PARylation, in a feed-forward loop, accelerates neuronal cell death by rendering a-synuclein more toxic. In addition, Puentes et al. [110] demonstrated that PAR interacts with phosphorylated α-Synuclein (pαSyn) in post-mortem brain samples of PD, Parkinson’s Disease dementia (PDD) and multiple system atrophy (MSA) patients, thus providing direct evidence for a role of PARP-1 and PARylation in the progression and severity of synucleinopathies. In ataxia telangiectasia mouse and *C. elegans* models, the levels of PARylation are increased, and the mitochondria result is significantly dysfunctional [111]. Similarly, overactivation of PARP-1 has been shown in other neurodegenerative diseases, such as HD [112], ALS [113,114] and multiple sclerosis [115].

In the brain of patients with Alzheimer’s disease, extensive PARP-1 activation is considered an early and important event of the pathogenesis [10]. PARP-1 overactivation is mainly related to the generation of reactive oxygen species (ROS) by β-amyloid aggregates which might depend on both the damaged function of the electron transport chain and/or on metals redox reaction (Cu^2+^, Zn^2+^ and Fe^2+^) involving Aβ species [116]. The accumulation of misfolded proteins may cause the functional decline of the mitochondria through bioenergetic defects, apoptosis, or autophagy [117,118,119].

Aβ accumulation activates PARP-1 protein specifically in astrocytes, leading indirectly to neuronal cell death [9]. Activated PARP-1 colocalizes with Aβ, tau and microtubule-associated protein 2 [12] and acts by promoting both the Aβ deposition and the formation of tau tangles that, in turn, aggravate the symptoms of AD [51]. Hyperactivated PARP-1 also promotes the DNA binding of NFkB in microglia [120], and Aβ-induced microglial activation is mitigated in mice mutant for PARP-1 [34]. Collectively, these findings support a crucial role of PARP-1 in the pathogenesis of AD and strongly suggest that targeting PARP-1 could be a promising strategy for minimizing the detrimental effects correlated with PARP-1 overactivation in AD.

Currently some PARP-1 inhibitors, including olaparib, rucaparib and niraparib, are approved as a therapy for several cancer types due to their synthetic lethality mechanism [121,122]. Repurposing of already approved PARP-1 inhibitors for pathological conditions characterized by PARP-1 hyperactivity could be an attractive strategy, since it may offer accelerated and cost-effective therapeutic opportunities for different neurodegenerative diseases, including AD.

Several previous studies indicate that PARP-1 inhibition could attenuate neurodegeneration and delay the progression of AD by preventing oxidative damage, mitochondrial impairment and neuroinflammation [35,123,124,125].

Nevertheless, most of them rely on in vitro cellular models that cannot capture the inherent complexity of a living organism.

In this study, taking advantage of AD transgenic fly models, we provided functional evidence that PARP-1 pharmacological inhibition can exert in vivo neuroprotective effects which might be helpful in preventing and treating AD symptoms.

In particular, we took advantage of two different genetically engineered *Drosophila* models; in the APP/BACE1 model, transgenic flies expressing both human APP and β-secretase BACE1 in the nervous system recapitulate the amyloidogenic proteolytic processing of APP by β- and γ-secretase, respectively, leading to the production of Aβ [71]. In the model Aβ42, transgenic flies overexpress a pan-neuronally secreted form of the human Aβ42 peptide, allowing them to bypass the side effects of the amyloid precursor processing [72]. Our results showed that both PARP inhibitors olaparib and MC2050 partially rescue the short lifespan of *elav-Gal4* > *APP*, *BACE1* flies, as well as significantly improve the impaired locomotor activity of *elav-Gal4* > *Aβ42* flies. In particular, the locomotor performance of *elav-Gal4* > *Aβ42* models increased when the flies were treated with 100 μM of MC2050, while the same concentration had no positive effect on the vitality of *elav-Gal4* > *APP*, *BACE1* models. On the other hand, the latter showed a positive response in terms of lifespan when treated with a concentration of 50 μM. This difference in drug efficiency could be due to the implicit diversity of the two models related to the severity of pathological phenotype.

The climbing and lifespan data obtained with PARP-1 inhibitors were further confirmed by RNAi-mediated genetic knockdown of PARP-1 in AD genetic background. Quantitative analysis of NAD^+^ cellular levels suggests that the phenotypic recovery obtained in lifespan and climbing assays could be related to an improvement in bioenergetic metabolism promoted by the inhibition of PARP-1, which favors the restoration of NAD^+^ concentrations.

Moreover, consistent with the results obtained in lifespan and climbing experiments, PARP-1 inhibition also leads to a strong reduction of Aβ42-positive puncta in adult brains from *elav-Gal4* > *Aβ42* flies. Since treatments with inhibitors do not impair the transcription of the Aβ42 pathogenic construct, we hypothesized that the effects of olaparib or MC2050 might be due to an increase of Aβ42 clearance or decreased Aβ42 toxic oligomers self-aggregation. The latter possibility is supported by recent papers showing that PARylation causes liquid demixing of intrinsically disordered proteins, accelerating their aggregation under pathological conditions [109,126].

Growing evidence suggests a detrimental effect of TEs activity in neurodegenerative disorders including AD [95,97]. In *Drosophila* models of human tauopathy, heterochromatin decondensation contributes to aberrant transposable element transcription [95,127,128]. Given that PARP-1 can substantially alter chromatin structure [129], we investigated whether PARP-1 inhibitors were capable of suppressing AD-induced transposable element dysregulation. The expression profiles of opus, copia, roo, springer and Aurora were analyzed in adult heads from *elav-Gal4* > *Aβ42* flies treated with or without PARP-1 inhibitors. PARP-1 inhibition exerted significant TEs downregulation, suggesting that its capability in suppressing AD-induced TEs activation might reside in the restoration of a more condensed chromatin on TE sequences. Tulin and coworkers also demonstrated that PARP-1 homozygous mutant larvae and adults exhibit significant changes in the expression of several TEs, suggesting that PARP-1 may be effectively involved in regulating the expression of mobile elements [63,130].

Several additional reports showed that TEs are modulated by histone modifications in complex patterns [131] and that PARP-1 is able to directly interact with nucleosomal core histones that, in turn, trigger PARP-1 enzymatic activity through their N-terminal domain modifications [132]. In addition, different authors also highlighted the potential functional relation between the activation of PARP-1 and the phosphorylation of the histone variant H2AX (γ-H2AX), normally observed in the presence of DNA damage [133,134]. Moreover, it has been observed that PARP-1 modulates the activity of histone-modifying enzymes [24,135,136]. Considering that, we determined the protein levels of the main histone post-translational modifications (PTM) involved in chromatin repression or activation such as H3K27me3, H3K9me3 and H3 pan-acetylated. In particular, a decrease in H3 pan-acetylation, H3K9me3 and H3K27me3 levels is shown in the *elav-Gal4* > *Aβ42* genotype treated with both PARP-1 inhibitors. The decrease of H3K9me3 induced by PARP-1 inhibition is in agreement with Bartlett et al. [137]. The authors showed that the competition between PARP-1/HPF1 (eponymous Histone PARylation Factor 1) complex and histone acetyltransferase leads to the mutual exclusion between the PARylation of H3S10 and H3K9ac, which is the PTM expected when the H3K9me3 is reduced. The overall results demonstrate that PARP-1 is able to modulate histone PTM profiles through its intrinsic enzyme activity to direct transcription [24] or structural component of chromatin [138].

Collectively, our data strongly suggest that PARP-1 inhibitors exert neuroprotective effects in AD through two different mechanisms. On the one hand, functional inhibition of PARP-1 antagonizes the aggregation of toxic Aβ oligomers; on the other hand, it partially suppresses AD-induced transposable element activation by epigenetically regulating chromatin structure and function. Our findings thus provide molecular mechanisms underlying neuroprotective effects of PARP-1 inhibitors and strengthen existing evidence for the potential therapeutic repurposing of PARP-inhibitors for the therapy of neurodegenerative disorders [121,139,140,141].

## Data Availability

Not applicable.

## Supplementary Materials

The following supporting information can be downloaded at: https://www.mdpi.com/article/10.3390/cells11081284/s1, Figure S1: Quantitative analysis of transcript levels of PARP-1 in Aβ42 and APP/BACE1 fly models; Figure S2: Quantitative analysis of climbing defects in AD fly models.

## References

[1] Braak H., Del Tredici K. (2015). Neuroanatomy and pathology of sporadic Alzheimer’s disease. Adv. Anat. Embryol. Cell Biology.

[2] Butterfield D.A. (2002). Amyloid beta-peptide (1-42)-induced oxidative stress and neurotoxicity: Implications for neurodegeneration in Alzheimer’s disease brain. A review. Free Radic. Res..

[3] Hayashi S., Sato N., Yamamoto A., Ikegame Y., Nakashima S., Ogihara T., Morishita R. (2009). Alzheimer disease-associated peptide, amyloid beta40, inhibits vascular regeneration with induction of endothelial autophagy. Arterioscler. Thromb. Vasc. Biol..

[4] Liu X., Jiao B., Shen L. (2018). The Epigenetics of Alzheimer’s Disease: Factors and Therapeutic Implications. Front. Genet..

[5] Luscher B., Ahel I., Altmeyer M., Ashworth A., Bai P., Chang P., Cohen M., Corda D., Dantzer F., Daugherty M.D. (2021). ADP-ribosyltransferases, an update on function and nomenclature. FEBS J.

[6] Love S., Barber R., Wilcock G.K. (1999). Increased poly(ADP-ribosyl)ation of nuclear proteins in Alzheimer’s disease. Brain.

[7] Strosznajder J.B., Jesko H., Strosznajder R.P. (2000). Effect of amyloid beta peptide on poly(ADP-ribose) polymerase activity in adult and aged rat hippocampus. Acta Biochim. Pol..

[8] Liu H.P., Lin W.Y., Wu B.T., Liu S.H., Wang W.F., Tsai C.H., Lee C.C., Tsai F.J. (2010). Evaluation of the poly(ADP-ribose) polymerase-1 gene variants in Alzheimer’s disease. J. Clin. Lab. Anal..

[9] Abeti R., Duchen M.R. (2012). Activation of PARP by oxidative stress induced by beta-amyloid: Implications for Alzheimer’s disease. Neurochem. Res..

[10] Strosznajder J.B., Czapski G.A., Adamczyk A., Strosznajder R.P. (2012). Poly(ADP-ribose) polymerase-1 in amyloid beta toxicity and Alzheimer’s disease. Mol. Neurobiol..

[11] Zeng J., Libien J., Shaik F., Wolk J., Hernandez A.I. (2016). Nucleolar PARP-1 Expression Is Decreased in Alzheimer’s Disease: Consequences for Epigenetic Regulation of rDNA and Cognition. Neural Plast..

[12] Narne P., Pandey V., Simhadri P.K., Phanithi P.B. (2017). Poly(ADP-ribose)polymerase-1 hyperactivation in neurodegenerative diseases: The death knell tolls for neurons. Semin. Cell Dev. Biol..

[13] Regier M., Liang J., Choi A., Verma K., Libien J., Hernandez A.I. (2019). Evidence for Decreased Nucleolar PARP-1 as an Early Marker of Cognitive Impairment. Neural Plast..

[14] Liu C., Fang Y. (2019). New insights of poly(ADP-ribosylation) in neurodegenerative diseases: A focus on protein phase separation and pathologic aggregation. Biochem. Pharmacol..

[15] Salech F., Ponce D.P., Paula-Lima A.C., SanMartin C.D., Behrens M.I. (2020). Nicotinamide, a Poly [ADP-Ribose] Polymerase 1 (PARP-1) Inhibitor, as an Adjunctive Therapy for the Treatment of Alzheimer’s Disease. Front. Aging Neurosci..

[16] D’Amours D., Desnoyers S., D’Silva I., Poirier G.G. (1999). Poly(ADP-ribosyl)ation reactions in the regulation of nuclear functions. Biochem. J..

[17] Kraus W.L. (2008). Transcriptional control by PARP-1: Chromatin modulation, enhancer-binding, coregulation, and insulation. Curr. Opin. Cell. Biol..

[18] Poirier G.G., de Murcia G., Jongstra-Bilen J., Niedergang C., Mandel P. (1982). Poly(ADP-ribosyl)ation of polynucleosomes causes relaxation of chromatin structure. Proc. Natl. Acad. Sci. USA.

[19] d’Erme M., Yang G., Sheagly E., Palitti F., Bustamante C. (2001). Effect of poly(ADP-ribosyl)ation and Mg2+ ions on chromatin structure revealed by scanning force microscopy. Biochemistry.

[20] Messner S., Altmeyer M., Zhao H., Pozivil A., Roschitzki B., Gehrig P., Rutishauser D., Huang D., Caflisch A., Hottiger M.O. (2010). PARP1 ADP-ribosylates lysine residues of the core histone tails. Nucleic Acids Res..

[21] Yang G., Chen Y., Wu J., Chen S.H., Liu X., Singh A.K., Yu X. (2020). Poly(ADP-ribosyl)ation mediates early phase histone eviction at DNA lesions. Nucleic Acids Res..

[22] Tulin A., Spradling A. (2003). Chromatin loosening by poly(ADP)-ribose polymerase (PARP) at Drosophila puff loci. Science.

[23] Happel N., Doenecke D. (2009). Histone H1 and its isoforms: Contribution to chromatin structure and function. Genes.

[24] Krishnakumar R., Kraus W.L. (2010). PARP-1 regulates chromatin structure and transcription through a KDM5B-dependent pathway. Mol. Cell.

[25] Krishnakumar R., Gamble M.J., Frizzell K.M., Berrocal J.G., Kininis M., Kraus W.L. (2008). Reciprocal binding of PARP-1 and histone H1 at promoters specifies transcriptional outcomes. Science.

[26] Halappanavar S.S., Shah G.M. (2004). Defective control of mitotic and post-mitotic checkpoints in poly(ADP-ribose) polymerase-1(-/-) fibroblasts after mitotic spindle disruption. Cell Cycle.

[27] Bai P., Canto C. (2012). The role of PARP-1 and PARP-2 enzymes in metabolic regulation and disease. Cell Metab..

[28] De Vos M., Schreiber V., Dantzer F. (2012). The diverse roles and clinical relevance of PARPs in DNA damage repair: Current state of the art. Biochem. Pharmacol..

[29] Burkle A., Virag L. (2013). Poly(ADP-ribose): PARadigms and PARadoxes. Mol. Asp. Med..

[30] Cho-Park P.F., Steller H. (2013). Proteasome regulation by ADP-ribosylation. Cell.

[31] Gupte R., Liu Z., Kraus W.L. (2017). PARPs and ADP-ribosylation: Recent advances linking molecular functions to biological outcomes. Genes Dev..

[32] Pascal J.M. (2018). The comings and goings of PARP-1 in response to DNA damage. DNA Repair.

[33] Abeti R., Abramov A.Y., Duchen M.R. (2011). Beta-amyloid activates PARP causing astrocytic metabolic failure and neuronal death. Brain.

[34] Kauppinen T.M., Suh S.W., Higashi Y., Berman A.E., Escartin C., Won S.J., Wang C., Cho S.H., Gan L., Swanson R.A. (2011). Poly(ADP-ribose)polymerase-1 modulates microglial responses to amyloid beta. J. Neuroinflammation.

[35] Martire S., Fuso A., Rotili D., Tempera I., Giordano C., De Zottis I., Muzi A., Vernole P., Graziani G., Lococo E. (2013). PARP-1 modulates amyloid beta peptide-induced neuronal damage. PLoS ONE.

[36] Bayrakdar E.T., Armagan G., Uyanikgil Y., Kanit L., Koylu E., Yalcin A. (2014). Ex vivo protective effects of nicotinamide and 3-aminobenzamide on rat synaptosomes treated with Abeta(1-42). Cell Biochem. Funct..

[37] Wencel P.L., Lukiw W.J., Strosznajder J.B., Strosznajder R.P. (2018). Inhibition of Poly(ADP-ribose) Polymerase-1 Enhances Gene Expression of Selected Sirtuins and APP Cleaving Enzymes in Amyloid Beta Cytotoxicity. Mol. Neurobiol..

[38] Alano C.C., Garnier P., Ying W., Higashi Y., Kauppinen T.M., Swanson R.A. (2010). NAD+ depletion is necessary and sufficient for poly(ADP-ribose) polymerase-1-mediated neuronal death. J. Neurosci..

[39] Correani V., Martire S., Mignogna G., Caruso L.B., Tempera I., Giorgi A., Grieco M., Mosca L., Schinina M.E., Maras B. (2019). Poly(ADP-ribosylated) proteins in beta-amyloid peptide-stimulated microglial cells. Biochem. Pharmacol..

[40] Fehr A.R., Singh S.A., Kerr C.M., Mukai S., Higashi H., Aikawa M. (2020). The impact of PARPs and ADP-ribosylation on inflammation and host-pathogen interactions. Genes. Dev..

[41] Jang S., Kim E.W., Zhang Y., Lee J., Cho S.Y., Ha J., Kim H., Kim E. (2018). Particulate matter increases beta-amyloid and activated glial cells in hippocampal tissues of transgenic Alzheimer’s mouse: Involvement of PARP-1. Biochem. Biophys. Res. Commun..

[42] Ke Y., Wang C., Zhang J., Zhong X., Wang R., Zeng X., Ba X. (2019). The Role of PARPs in Inflammation-and Metabolic-Related Diseases: Molecular Mechanisms and Beyond. Cells.

[43] Maiuri T., Bowie L.E., Truant R. (2019). DNA Repair Signaling of Huntingtin: The Next Link Between Late-Onset Neurodegenerative Disease and Oxidative DNA Damage. DNA Cell Biol..

[44] McGurk L., Gomes E., Guo L., Mojsilovic-Petrovic J., Tran V., Kalb R.G., Shorter J., Bonini N.M. (2018). Poly(ADP-Ribose) Prevents Pathological Phase Separation of TDP-43 by Promoting Liquid Demixing and Stress Granule Localization. Mol. Cell.

[45] Morris G., Walker A.J., Berk M., Maes M., Puri B.K. (2018). Cell Death Pathways: A Novel Therapeutic Approach for Neuroscientists. Mol. Neurobiol..

[46] Vida A., Marton J., Miko E., Bai P. (2017). Metabolic roles of poly(ADP-ribose) polymerases. Semin. Cell. Dev. Biol..

[47] Schneider L.S. (2014). Rethinking the Food and Drug Administration’s 2013 guidance on developing drugs for early-stage Alzheimer’s disease. Alzheimer’s Dement..

[48] Peskind E.R., Potkin S.G., Pomara N., Ott B.R., Graham S.M., Olin J.T., McDonald S. (2006). Memantine treatment in mild to moderate Alzheimer disease: A 24-week randomized, controlled trial. Am. J. Geriatr. Psychiatry.

[49] Cummings J., Reiber C., Kumar P. (2018). The price of progress: Funding and financing Alzheimer’s disease drug development. Alzheimer’s Dement..

[50] Sevigny J., Chiao P., Bussiere T., Weinreb P.H., Williams L., Maier M., Dunstan R., Salloway S., Chen T., Ling Y. (2016). The antibody aducanumab reduces Abeta plaques in Alzheimer’s disease. Nature.

[51] Martire S., Mosca L., d’Erme M. (2015). PARP-1 involvement in neurodegeneration: A focus on Alzheimer’s and Parkinson’s diseases. Mech. Ageing Dev..

[52] Marin E.C., Jefferis G.S., Komiyama T., Zhu H., Luo L. (2002). Representation of the glomerular olfactory map in the Drosophila brain. Cell.

[53] Rein K., Zockler M., Mader M.T., Grubel C., Heisenberg M. (2002). The Drosophila standard brain. Curr. Biol..

[54] Wong A.M., Wang J.W., Axel R. (2002). Spatial representation of the glomerular map in the Drosophila protocerebrum. Cell.

[55] Jeon Y., Lee J.H., Choi B., Won S.Y., Cho K.S. (2020). Genetic Dissection of Alzheimer’s Disease Using Drosophila Models. Int. J. Mol. Sci..

[56] Tan F.H.P., Azzam G. (2017). Drosophila melanogaster: Deciphering Alzheimer’s Disease. Malays. J. Med. Sci..

[57] Tsuda L., Lim Y.M. (2018). Alzheimer’s Disease Model System Using Drosophila. Adv. Exp. Med. Biol..

[58] Prussing K., Voigt A., Schulz J.B. (2013). Drosophila melanogaster as a model organism for Alzheimer’s disease. Mol. Neurodegener..

[59] Marsh J.L., Thompson L.M. (2006). Drosophila in the study of neurodegenerative disease. Neuron.

[60] Thomas C.J., Kotova E., Andrake M., Adolf-Bryfogle J., Glaser R., Regnard C., Tulin A.V. (2014). Kinase-mediated changes in nucleosome conformation trigger chromatin decondensation via poly(ADP-ribosyl)ation. Mol. Cell.

[61] Li N., Chen J. (2014). ADP-ribosylation: Activation, recognition, and removal. Mol. Cells.

[62] Ji Y., Tulin A.V. (2010). The roles of PARP1 in gene control and cell differentiation. Curr. Opin. Genet. Dev..

[63] Tulin A., Stewart D., Spradling A.C. (2002). The Drosophila heterochromatic gene encoding poly(ADP-ribose) polymerase (PARP) is required to modulate chromatin structure during development. Genes Dev..

[64] Gunderson C.C., Moore K.N. (2015). Olaparib: An oral PARP-1 and PARP-2 inhibitor with promising activity in ovarian cancer. Future Oncol..

[65] Paik J. (2021). Olaparib: A Review as First-Line Maintenance Therapy in Advanced Ovarian Cancer. Target. Oncol..

[66] Mosca L., Rotili D., Tempera I., Masci A., Fontana M., Chiaraluce R., Mastromarino P., d’Erme M., Mai A. (2011). Biological effects of MC2050, a quinazoline-based PARP-1 inhibitor, in human neuroblastoma and EBV-positive Burkitt’s lymphoma cells. ChemMedChem.

[67] Menear K.A., Adcock C., Boulter R., Cockcroft X.L., Copsey L., Cranston A., Dillon K.J., Drzewiecki J., Garman S., Gomez S. (2008). 4-[3-(4-cyclopropanecarbonylpiperazine-1-carbonyl)-4-fluorobenzyl]-2H-phthalazin- 1-one: A novel bioavailable inhibitor of poly(ADP-ribose) polymerase-1. J. Med. Chem..

[68] Livak K.J., Schmittgen T.D. (2001). Analysis of relative gene expression data using real-time quantitative PCR and the 2(-Delta Delta C(T)) Method. Methods.

[69] Wu J.S., Luo L. (2006). A protocol for dissecting Drosophila melanogaster brains for live imaging or immunostaining. Nat. Protoc..

[70] Ali Y.O., Escala W., Ruan K., Zhai R.G. (2011). Assaying locomotor, learning, and memory deficits in Drosophila models of neurodegeneration. J. Vis. Exp..

[71] Chakraborty R., Vepuri V., Mhatre S.D., Paddock B.E., Miller S., Michelson S.J., Delvadia R., Desai A., Vinokur M., Melicharek D.J. (2011). Characterization of a Drosophila Alzheimer’s disease model: Pharmacological rescue of cognitive defects. PLoS ONE.

[72] Crowther D.C., Kinghorn K.J., Miranda E., Page R., Curry J.A., Duthie F.A., Gubb D.C., Lomas D.A. (2005). Intraneuronal Abeta, non-amyloid aggregates and neurodegeneration in a Drosophila model of Alzheimer’s disease. Neuroscience.

[73] Finelli A., Kelkar A., Song H.J., Yang H., Konsolaki M. (2004). A model for studying Alzheimer’s Abeta42-induced toxicity in Drosophila melanogaster. Mol. Cell. Neurosci..

[74] Iijima K., Liu H.P., Chiang A.S., Hearn S.A., Konsolaki M., Zhong Y. (2004). Dissecting the pathological effects of human Abeta40 and Abeta42 in Drosophila: A potential model for Alzheimer’s disease. Proc. Natl. Acad. Sci. USA.

[75] Greenwald S.H., Brown E.E., Scandura M.J., Hennessey E., Farmer R., Du J., Wang Y., Pierce E.A. (2021). Mutant Nmnat1 leads to a retina-specific decrease of NAD+ accompanied by increased poly(ADP-ribose) in a mouse model of NMNAT1-associated retinal degeneration. Hum. Mol. Genet..

[76] Tiraboschi P., Sabbagh M.N., Hansen L.A., Salmon D.P., Merdes A., Gamst A., Masliah E., Alford M., Thal L.J., Corey-Bloom J. (2004). Alzheimer disease without neocortical neurofibrillary tangles: “a second look”. Neurology.

[77] Wang Z., Yang L., Zheng H. (2012). Role of APP and Abeta in synaptic physiology. Curr. Alzheimer Res..

[78] Wolfe M.S. (2012). Processive proteolysis by gamma-secretase and the mechanism of Alzheimer’s disease. Biol. Chem..

[79] Hardy J., Selkoe D.J. (2002). The amyloid hypothesis of Alzheimer’s disease: Progress and problems on the road to therapeutics. Science.

[80] Karran E., Mercken M., De Strooper B. (2011). The amyloid cascade hypothesis for Alzheimer’s disease: An appraisal for the development of therapeutics. Nat. Rev. Drug Discov..

[81] Iijima K., Chiang H.C., Hearn S.A., Hakker I., Gatt A., Shenton C., Granger L., Leung A., Iijima-Ando K., Zhong Y. (2008). Abeta42 mutants with different aggregation profiles induce distinct pathologies in Drosophila. PLoS ONE.

[82] Jonsson M.E., Garza R., Johansson P.A., Jakobsson J. (2020). Transposable Elements: A Common Feature of Neurodevelopmental and Neurodegenerative Disorders. Trends Genet..

[83] Saleh A., Macia A., Muotri A.R. (2019). Transposable Elements, Inflammation, and Neurological Disease. Front. Neurol..

[84] Tam C., Wong J.H., Ng T.B., Tsui S.K.W., Zuo T. (2019). Drugs for Targeted Therapies of Alzheimer’s Disease. Curr. Med. Chem..

[85] Muotri A.R., Marchetto M.C., Coufal N.G., Oefner R., Yeo G., Nakashima K., Gage F.H. (2010). L1 retrotransposition in neurons is modulated by MeCP2. Nature.

[86] Coufal N.G., Garcia-Perez J.L., Peng G.E., Marchetto M.C., Muotri A.R., Mu Y., Carson C.T., Macia A., Moran J.V., Gage F.H. (2011). Ataxia telangiectasia mutated (ATM) modulates long interspersed element-1 (L1) retrotransposition in human neural stem cells. Proc. Natl. Acad. Sci. USA.

[87] Kaneko H., Dridi S., Tarallo V., Gelfand B.D., Fowler B.J., Cho W.G., Kleinman M.E., Ponicsan S.L., Hauswirth W.W., Chiodo V.A. (2011). DICER1 deficit induces Alu RNA toxicity in age-related macular degeneration. Nature.

[88] Lathe R., Harris A. (2009). Differential display detects host nucleic acid motifs altered in scrapie-infected brain. J. Mol. Biol..

[89] Bundo M., Toyoshima M., Okada Y., Akamatsu W., Ueda J., Nemoto-Miyauchi T., Sunaga F., Toritsuka M., Ikawa D., Kakita A. (2014). Increased l1 retrotransposition in the neuronal genome in schizophrenia. Neuron.

[90] Blaudin de The F.X., Rekaik H., Peze-Heidsieck E., Massiani-Beaudoin O., Joshi R.L., Fuchs J., Prochiantz A. (2018). Engrailed homeoprotein blocks degeneration in adult dopaminergic neurons through LINE-1 repression. EMBO J..

[91] Krug L., Chatterjee N., Borges-Monroy R., Hearn S., Liao W.W., Morrill K., Prazak L., Rozhkov N., Theodorou D., Hammell M. (2017). Retrotransposon activation contributes to neurodegeneration in a Drosophila TDP-43 model of ALS. PLoS Genet..

[92] Li W., Jin Y., Prazak L., Hammell M., Dubnau J. (2012). Transposable elements in TDP-43-mediated neurodegenerative disorders. PLoS ONE.

[93] Romano G., Klima R., Feiguin F. (2020). TDP-43 prevents retrotransposon activation in the Drosophila motor system through regulation of Dicer-2 activity. BMC Biol..

[94] Casale A.M., Liguori F., Ansaloni F., Cappucci U., Finaurini S., Spirito G., Persichetti F., Sanges R., Gustincich S., Piacentini L. (2022). Transposable element activation promotes neurodegeneration in a Drosophila model of Huntington’s disease. iScience.

[95] Guo C., Jeong H.H., Hsieh Y.C., Klein H.U., Bennett D.A., De Jager P.L., Liu Z., Shulman J.M. (2018). Tau Activates Transposable Elements in Alzheimer’s Disease. Cell Rep..

[96] Misiak B., Ricceri L., Sasiadek M.M. (2019). Transposable Elements and Their Epigenetic Regulation in Mental Disorders: Current Evidence in the Field. Front. Genet..

[97] Sun W., Samimi H., Gamez M., Zare H., Frost B. (2018). Pathogenic tau-induced piRNA depletion promotes neuronal death through transposable element dysregulation in neurodegenerative tauopathies. Nat. Neurosci..

[98] Malik N., Smulson M. (1984). A relationship between nuclear poly(adenosine diphosphate ribosylation) and acetylation posttranslational modifications. 1. Nucleosome studies. Biochemistry.

[99] Cohen-Armon M., Visochek L., Rozensal D., Kalal A., Geistrikh I., Klein R., Bendetz-Nezer S., Yao Z., Seger R. (2007). DNA-independent PARP-1 activation by phosphorylated ERK2 increases Elk1 activity: A link to histone acetylation. Mol. Cell.

[100] Chen H., Ruiz P.D., Novikov L., Casill A.D., Park J.W., Gamble M.J. (2014). MacroH2A1.1 and PARP-1 cooperate to regulate transcription by promoting CBP-mediated H2B acetylation. Nat. Struct. Mol. Biol..

[101] Martin K.A., Cesaroni M., Denny M.F., Lupey L.N., Tempera I. (2015). Global Transcriptome Analysis Reveals That Poly(ADP-Ribose) Polymerase 1 Regulates Gene Expression through EZH2. Mol. Cell. Biol..

[102] Greeve I., Kretzschmar D., Tschape J.A., Beyn A., Brellinger C., Schweizer M., Nitsch R.M., Reifegerste R. (2004). Age-dependent neurodegeneration and Alzheimer-amyloid plaque formation in transgenic Drosophila. J. Neurosci..

[103] Martire S., Fuso A., Mosca L., Forte E., Correani V., Fontana M., Scarpa S., Maras B., d’Erme M. (2016). Bioenergetic Impairment in Animal and Cellular Models of Alzheimer’s Disease: PARP-1 Inhibition Rescues Metabolic Dysfunctions. J. Alzheimer’s. Dis..

[104] Leung A.K.L. (2017). PARPs. Curr. Biol..

[105] Beneke S., Burkle A. (2004). Poly(ADP-ribosyl)ation, PARP, and aging. Sci. Aging Knowl. Environ..

[106] Kauppinen T.M., Swanson R.A. (2007). The role of poly(ADP-ribose) polymerase-1 in CNS disease. Neuroscience.

[107] Alhosaini K., Ansari M.A., Nadeem A., Bakheet S.A., Attia S.M., Alhazzani K., Albekairi T.H., Al-Mazroua H.A., Mahmood H.M., Ahmad S.F. (2021). 5-Aminoisoquinolinone, a PARP-1 Inhibitor, Ameliorates Immune Abnormalities through Upregulation of Anti-Inflammatory and Downregulation of Inflammatory Parameters in T Cells of BTBR Mouse Model of Autism. Brain Sci..

[108] Mao K., Zhang G. (2021). The role of PARP1 in neurodegenerative diseases and aging. FEBS J..

[109] Kam T.I., Mao X., Park H., Chou S.C., Karuppagounder S.S., Umanah G.E., Yun S.P., Brahmachari S., Panicker N., Chen R. (2018). Poly(ADP-ribose) drives pathologic alpha-synuclein neurodegeneration in Parkinson’s disease. Science.

[110] Puentes L.N., Lengyel-Zhand Z., Lee J.Y., Hsieh C.J., Schneider M.E., Edwards K.J., Luk K.C., Lee V.M., Trojanowski J.Q., Mach R.H. (2021). Poly (ADP-ribose) Interacts With Phosphorylated alpha-Synuclein in Post Mortem PD Samples. Front. Aging Neurosci..

[111] Fang E.F., Kassahun H., Croteau D.L., Scheibye-Knudsen M., Marosi K., Lu H., Shamanna R.A., Kalyanasundaram S., Bollineni R.C., Wilson M.A. (2016). NAD(+) Replenishment Improves Lifespan and Healthspan in Ataxia Telangiectasia Models via Mitophagy and DNA Repair. Cell Metab..

[112] Cardinale A., Paldino E., Giampa C., Bernardi G., Fusco F.R. (2015). PARP-1 Inhibition Is Neuroprotective in the R6/2 Mouse Model of Huntington’s Disease. PLoS ONE.

[113] Rulten S.L., Rotheray A., Green R.L., Grundy G.J., Moore D.A., Gomez-Herreros F., Hafezparast M., Caldecott K.W. (2014). PARP-1 dependent recruitment of the amyotrophic lateral sclerosis-associated protein FUS/TLS to sites of oxidative DNA damage. Nucleic Acids Res..

[114] Wang H., Guo W., Mitra J., Hegde P.M., Vandoorne T., Eckelmann B.J., Mitra S., Tomkinson A.E., Van Den Bosch L., Hegde M.L. (2018). Mutant FUS causes DNA ligation defects to inhibit oxidative damage repair in Amyotrophic Lateral Sclerosis. Nat. Commun..

[115] Farez M.F., Quintana F.J., Gandhi R., Izquierdo G., Lucas M., Weiner H.L. (2009). Toll-like receptor 2 and poly(ADP-ribose) polymerase 1 promote central nervous system neuroinflammation in progressive EAE. Nat. Immunol..

[116] Tillement L., Lecanu L., Papadopoulos V. (2011). Alzheimer’s disease: Effects of beta-amyloid on mitochondria. Mitochondrion.

[117] Chaturvedi R.K., Beal M.F. (2013). Mitochondria targeted therapeutic approaches in Parkinson’s and Huntington’s diseases. Mol. Cell. Neurosci..

[118] Ghavami S., Shojaei S., Yeganeh B., Ande S.R., Jangamreddy J.R., Mehrpour M., Christoffersson J., Chaabane W., Moghadam A.R., Kashani H.H. (2014). Autophagy and apoptosis dysfunction in neurodegenerative disorders. Prog. Neurobiol..

[119] Kesidou E., Lagoudaki R., Touloumi O., Poulatsidou K.N., Simeonidou C. (2013). Autophagy and neurodegenerative disorders. Neural. Regen. Res..

[120] Chiarugi A., Moskowitz M.A. (2003). Poly(ADP-ribose) polymerase-1 activity promotes NF-kappaB-driven transcription and microglial activation: Implication for neurodegenerative disorders. J. Neurochem..

[121] Berger N.A., Besson V.C., Boulares A.H., Burkle A., Chiarugi A., Clark R.S., Curtin N.J., Cuzzocrea S., Dawson T.M., Dawson V.L. (2018). Opportunities for the repurposing of PARP inhibitors for the therapy of non-oncological diseases. Br. J. Pharmacol..

[122] Bitler B.G., Watson Z.L., Wheeler L.J., Behbakht K. (2017). PARP inhibitors: Clinical utility and possibilities of overcoming resistance. Gynecol. Oncol..

[123] Zeng K.W., Wang X.M., Ko H., Kwon H.C., Cha J.W., Yang H.O. (2011). Hyperoside protects primary rat cortical neurons from neurotoxicity induced by amyloid beta-protein via the PI3K/Akt/Bad/Bcl(XL)-regulated mitochondrial apoptotic pathway. Eur. J. Pharm..

[124] Czapski G.A., Cieslik M., Wencel P.L., Wojtowicz S., Strosznajder R.P., Strosznajder J.B. (2018). Inhibition of poly(ADP-ribose) polymerase-1 alters expression of mitochondria-related genes in PC12 cells: Relevance to mitochondrial homeostasis in neurodegenerative disorders. Biochim. Biophys. Acta Mol. Cell Res..

[125] Gao C.Z., Dong W., Cui Z.W., Yuan Q., Hu X.M., Wu Q.M., Han X., Xu Y., Min Z.L. (2019). Synthesis, preliminarily biological evaluation and molecular docking study of new Olaparib analogues as multifunctional PARP-1 and cholinesterase inhibitors. J. Enzym. Inhib. Med. Chem..

[126] Altmeyer M., Neelsen K.J., Teloni F., Pozdnyakova I., Pellegrino S., Grofte M., Rask M.D., Streicher W., Jungmichel S., Nielsen M.L. (2015). Liquid demixing of intrinsically disordered proteins is seeded by poly(ADP-ribose). Nat. Commun..

[127] Thomas E.O., Ramirez P., Hyman B.T., Ray W.J., Frost B. (2021). Testing the neuroinflammatory role of tau-induced transposable elements in tauopathy. Alzheimer’s Dement..

[128] Frost B., Hemberg M., Lewis J., Feany M.B. (2014). Tau promotes neurodegeneration through global chromatin relaxation. Nat. Neurosci..

[129] Quenet D., El Ramy R., Schreiber V., Dantzer F. (2009). The role of poly(ADP-ribosyl)ation in epigenetic events. Int. J. Biochem. Cell Biol..

[130] Bordet G., Lodhi N., Guo D., Kossenkov A., Tulin A.V. (2020). Poly(ADP-ribose) polymerase 1 in genome-wide expression control in Drosophila. Sci. Rep..

[131] He J., Fu X., Zhang M., He F., Li W., Abdul M.M., Zhou J., Sun L., Chang C., Li Y. (2019). Transposable elements are regulated by context-specific patterns of chromatin marks in mouse embryonic stem cells. Nat. Commun..

[132] Pinnola A., Naumova N., Shah M., Tulin A.V. (2007). Nucleosomal core histones mediate dynamic regulation of poly(ADP-ribose) polymerase 1 protein binding to chromatin and induction of its enzymatic activity. J. Biol. Chem..

[133] Kotova E., Lodhi N., Jarnik M., Pinnola A.D., Ji Y., Tulin A.V. (2011). Drosophila histone H2A variant (H2Av) controls poly(ADP-ribose) polymerase 1 (PARP1) activation in chromatin. Proc. Natl. Acad. Sci. USA.

[134] Sharma G.P., Gurung S.K., Inam A., Nigam L., Bist A., Mohapatra D., Senapati S., Subbarao N., Azam A., Mondal N. (2019). CID-6033590 inhibits p38MAPK pathway and induces S-phase cell cycle arrest and apoptosis in DU145 and PC-3 cells. Toxicol. Vitr..

[135] Caruso L.B., Martin K.A., Lauretti E., Hulse M., Siciliano M., Lupey-Green L.N., Abraham A., Skorski T., Tempera I. (2018). Poly(ADP-ribose) Polymerase 1, PARP1, modifies EZH2 and inhibits EZH2 histone methyltransferase activity after DNA damage. Oncotarget.

[136] Rudolph J., Roberts G., Luger K. (2021). Histone Parylation factor 1 contributes to the inhibition of PARP1 by cancer drugs. Nat. Commun..

[137] Bartlett E., Bonfiglio J.J., Prokhorova E., Colby T., Zobel F., Ahel I., Matic I. (2018). Interplay of Histone Marks with Serine ADP-Ribosylation. Cell Rep..

[138] Aguilar-Quesada R., Munoz-Gamez J.A., Martin-Oliva D., Peralta A., Valenzuela M.T., Matinez-Romero R., Quiles-Perez R., Menissier-de Murcia J., de Murcia G., Ruiz de Almodovar M. (2007). Interaction between ATM and PARP-1 in response to DNA damage and sensitization of ATM deficient cells through PARP inhibition. BMC Mol. Biol..

[139] Mekhaeil M., Dev K.K., Conroy M.J. (2022). Existing Evidence for the Repurposing of PARP-1 Inhibitors in Rare Demyelinating Diseases. Cancers.

[140] Puentes L.N., Lengyel-Zhand Z., Reilly S.W., Mach R.H. (2021). Evaluation of a Low-Toxicity PARP Inhibitor as a Neuroprotective Agent for Parkinson’s Disease. Mol. Neurobiol..

[141] Sahaboglu A., Miranda M., Canjuga D., Avci-Adali M., Savytska N., Secer E., Feria-Pliego J.A., Kayik G., Durdagi S. (2020). Drug repurposing studies of PARP inhibitors as a new therapy for inherited retinal degeneration. Cell. Mol. Life. Sci..

