# Supplementary material for: Neuroprotective Effects of PARP Inhibitors in Drosophila Models of Alzheimer’s Disease
Source: Cells. 2022 Apr 9;11(8):1284. doi: 10.3390/cells11081284 (PMC9027574; doi:10.3390/cells11081284)
Supplement: Supplementary file 1 [file cells-11-01284-s001.zip › cells-1589947-supplementary.pdf]

Supplementary Information

Neuroprotective effects of PARP inhibitors in *Drosophila* models of Alzheimer's disease

Anna Maggiore, Assunta Maria Casale, Walter Toscanelli, Ugo Cappucci, Dante Rotili, Maddalena Grieco, Jean-Philippe Gagné, Guy G. Poirier, Maria d'Erme, Lucia Piacentini

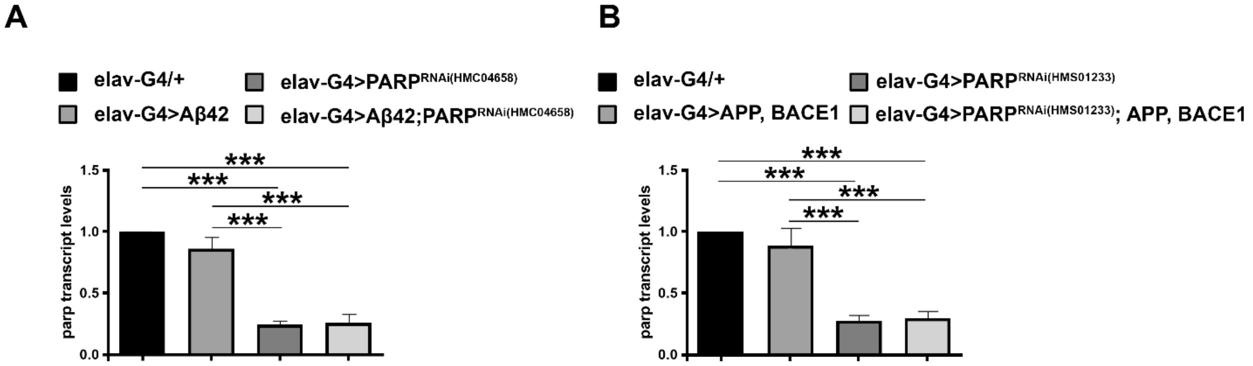

Supplementary Figure S1. Quantitative analysis of transcript levels of PARP-1 in Aβ42 and APP/BACE1 fly models. qRT-PCR analysis assessing PARP-1 mRNA levels in head tissues isolated from both Aβ42 (A) and APP/BACE1 (B) AD models following *in vivo* PARP-1 RNAi. The constitutive *rp49* was examined as an endogenous control. The data are expressed as "fold enrichment" with respect to *elav-Gal4/+* and represent the mean ± SEM of three independent experiments. Statistical significance was determined using the one-way ANOVA test followed by the Tukey test for multiple comparisons (***p<0.001).

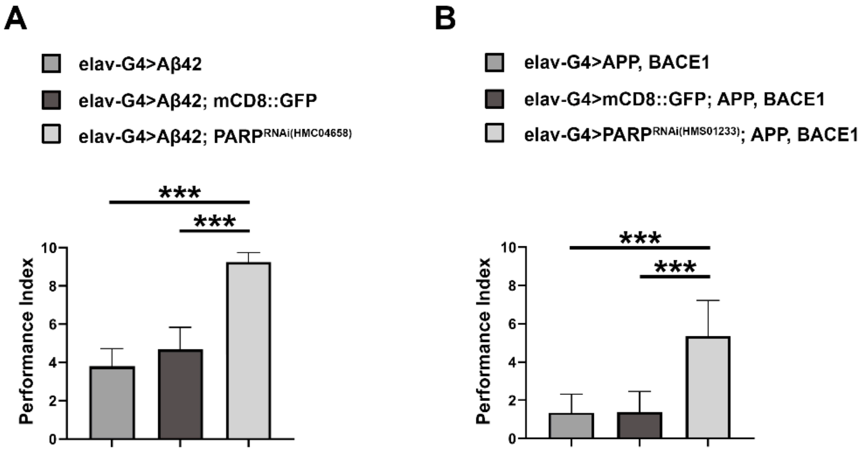

Supplementary Figure S2. Quantitative analysis of climbing defects in AD fly models. (A) Climbing test on *elav-Gal4> Aβ42*, *elav-Gal4> mCD8::GFP; Aβ42,* and *elav-Gal4>Aβ42; PARP-1*$^{RNAi(HMC04658)}$ flies. (B) Climbing test on *elav-Gal4> APP, BACE1*, *elav-Gal4> mCD8::GFP; APP, BACE1,* and *elav-Gal4> PARP-1*$^{RNAi(HMS01233)}$*; APP, BACE1* flies. Statistical significance was determined using the one-way ANOVA test followed by the Tukey test for multiple comparisons (***p<0.001).
